# The Need for Multidisciplinary Research within the History and Theory of Homeopathy *

**DOI:** 10.1055/s-0040-1714740

**Published:** 2020-09-08

**Authors:** Josef M. Schmidt

**Affiliations:** 1 Institute of Ethics, History, and Theory of Medicine, Ludwig Maximilian's University, Munich, Germany

**Keywords:** history of medicine, theory of medicine, homeopathy, history of science, theory of science

## Abstract

The controversial issue of homeopathy's scientificity will, in all probability, not be settled by means of clinical trials alone, as long as uncertainty or ignorance about methodological, philosophical, and socio-economical essentials prevail on both sides of the argument. Rather than uncritically adopt the standards of the currently predominant paradigm, homeopathy should not forget its roots, peculiarities, and self-conception. Contrary to conventional medicine, it is based on a teleological image of humanity, a holistic and sustainable approach towards curing sickness, and an up-to-date concept of medical theory in terms of healing arts. However, under today's frameworked conditions of industrialisation, commercialisation and commodification, the strengths of homeopathy tend to be disregarded or even attacked, and a special kind of reductionist and materialist rationality, compatible with expanding markets and profits, is preferably facilitated. To reveal and demonstrate these developments and relationships on a scientific level, there is a need for multidisciplinary research on the part of the humanities, such as history and theory of medicine, history and theory of science, history of economics, sociology of scientific knowledge, and philosophy.

## Introduction

As every crisis is also an opportunity, the present COVID-19 pandemic—by prompting governments all over the globe to impose drastic measures on their citizens, restricting civil rights and social, economic, and cultural life—provides ample stimulus to meditate on the relationship (and difference) between scientific knowledge and political action. Unsettled by the velocity and severity of unprecedented changes on many levels of their existence, more and more people are beginning to doubt whether natural or mathematical science alone may suffice to guide humanity through this crisis in the long run. They now prefer to ask for more perspectives, such as economic, social, psychological, educational, and cultural sciences and expertise, to be taken as an additional basis for sound informed decisions.

Homeopathy could take advantage of this recent opening of discussion towards a broader understanding of rationality and a renaissance of elementary questions, such as: ‘What do we really want, what is it that really matters in life, what price are we ready to pay?’. For long enough homeopathy has been assessed and decried merely on the basis of (wanting) statistical evidence, ignoring its other important dimensions regarding its place in the history, culture and science (in the broadest sense) of human civilisation.

Political as well as medical and administrative decisions should of course be taken on the basis of evidence—as far and broad as it may be available, i.e. preferably in its entirety. Hence, instead of relying exclusively on (always imperfect) epidemiologic numbers, assumptions and simulations, multidisciplinary collaboration of natural sciences with the humanities—especially historical, philosophical and cultural studies—may prove to be needed in coping with the challenge of epidemics, as well as in appraising homeopathy.

## Is Homeopathy a Medical Science?

One of the most undisputed statements among homeopaths may be the assertion that homeopathy is a medical science. This implies that it must first be considered as a medical discipline, i.e. as a part of medicine, and second as a scientific method, i.e. as a part of science. Since Hahnemann founded homeopathy as a rational and scientific medicine, this claim has become one of the most prominent features of homeopathy's corporate identity. Ironically, however, the charge most unanimously stated by critics and so-called sceptics is the imputation that homeopathy is not scientific and hence can not be a part of medical science. According to their argument, only conventional medicine is founded upon natural science, which has progressed tremendously since the days of Hahnemann, having established standards of verification procedures not met by homeopathy.

The usual reaction on the part of homeopaths to this seemingly devastating reproach by fundamentalists of natural science, and advocates of evidence-based medicine, is to try to disprove the accusation by demonstrating evidence of efficacy and effectiveness, by means of clinical studies, randomised controlled trials (RCTs), and the like. By doing so, however, they comply to their critics’ standards of proof and thus to the framework and definition of what, according to them, has to be considered as scientific medicine. However, hundreds of conducted clinical trials and observational studies published, with a considerable part indicating positive results for homeopathy, have not convinced scientific medicine hardliners. 1 2 In fact, systematic review of the homeopathy RCT literature shows that RCT findings collectively are somewhat inconclusive. 3 4 5

Far from seriously taking into consideration positive outcomes of studies on homeopathy, a new faction of critics, in particular Gorski and Novella, preferred to switch argumentation, indicating that even randomised double-blind placebo-controlled clinical studies of best quality may sometimes display positive results through coincidence, rendering them incapable of ever producing reliable evidence as to causality. To prevent homeopaths from fulfilling the criteria of evidence-based medicine, and thus becoming a part of the scientific community based on this concept, they suggested the creation of a stricter concept, called science-based medicine, according to which treatments, whose basic principles are not in accordance with established natural laws, should no longer be tested. 6

This is a rough summarisation of the deadlock situation, in which both homeopathy and conventional medicine are trapped, when homeopathy claims to be a science and conventional medicine denies it. This conflict, however, may not be irretrievable, but it results from an uncritical use and acceptance of problematic notions (such as medicine and science) and the equation of non-comparable subject-matters (such as homeopathy and conventional medicine). It rests on the presupposition of commonplace concepts of science and scientific medicine derived from the paradigm of natural and technical sciences, such as to be the methodical quest for natural laws by allegedly neutral observers or the like, and the widespread (even though outdated) assumption that medicine is an applied natural science. Only if this were true would both homeopathy and conventional medicine have to be checked and judged, verified or falsified, along these lines of quasi-absolute criteria and standards.

Nevertheless, as the history and theory of medicine may show, concepts of science and medicine are far from being unchangeable or absolute, but rather variable and dependent upon context and interests. 7 Obviously, the dominant present-day ideas about science and medicine, as underlying common medical practice, are uncritical and in favour of the scientificity of conventional medicine. Since under these presuppositions homeopaths come off relatively badly, they obviously have a vital reason for scrutinising and challenging this way of thinking. In fact, only by questioning the absoluteness of the standards of conventional medicine and by propounding an independent distinct methodology, may they be justified in considering homeopathy a practical scientific medicine. 8

## Homeopathy versus Conventional Medicine

On a phenomenological level, both conventional medicine and homeopathy are treating patients and are therefore medical therapies and, as such, part of medicine in the broadest sense. However, their differences are obvious and detectable on many levels, even from the standpoint of the patient. For example, in conventional medicine, as a rule, the doctor has very little time. In the consultation he or she focuses on so-called diagnoses, and before long prescribes for each diagnosis a separate medication. So the patient has to take, e.g. an analgesic against a headache, a tranquilliser against sleeplessness, a laxative against constipation, etc., and has to accept the risks of side-effects, as well as habituation, dependence or addiction, and the high costs of long-term therapy. In contrast, a homeopathic doctor, as a rule, interacts with the patient individually, asking considerably more questions, and finally prescribes one single remedy to treat all of the person's complaints simultaneously, often with a ‘one-off’ dose and usually without side-effects. The costs for the remedy, compared with those of the conventional medicine, are negligible.

These differences within real existing medical practice, verifiable by anybody, may demonstrate that the approach taken by conventional medicine is not self-evident, not singular, and not without alternative. On a closer, more systematic, look one may find that behind these differences in appearance there are two distinct philosophies based on premises antithetical and yet complementary to each other. They may each be summed up in seven points. 9

### Premises of Conventional Medicine, as Ordinarily Practised

Conventional drug therapy rests upon the following assumptions:

1. Every patient is a part of the species *Homo sapiens* —according to natural science's object being the general, not the individual.
2. The human body is composed of physical components interacting with each other—according to the doctrine of materialism (see Discussion).
3. These interactions are explainable in principle mechanically, physically, chemically, etc.—according to the doctrine of positivism of science (see Discussion).
4. Within the body, single sub-areas may be isolated and examined separately—according to the specialisation of science and conventional medicine.
5. Dysfunction of any part of the body means a derailment which has to be counteracted—in analogy to the malfunction of machines, where leakage has to be sealed, constrictions dilated, heated parts cooled, etc.
6. Drugs have to be applied in such doses that the effect will be as certain and uniform as possible with everybody. They must be reproducible.
7. The remainder of effects, so-called side-effects, have to be accepted, as there is no way to avoid them.

### Premises of Homeopathy, as Conceptualised by Hahnemann

In contrast, homeopathic therapeutics rest upon the following assumptions:

1. Every patient is a unique individual, and every illness is singular, distinctive, and unique.
2. Every individual is an organism, reacting to stimuli in a purposeful way; it is not a mechanism, but a self-acting subject.
3. The animating principle of the organism may be called life-force, vital principle, or the like; however, it cannot be explained by reductionistic natural sciences (see Discussion).
4. The living organism reacts to stimuli as a whole, as a unity, as there is just one vital force.
5. Dysfunctions in the life of the organism indicate a reaction of the same to disturbing stimuli and should therefore not be suppressed, but supported in principle, as long as the organism is not completely overwhelmed.
6. Remedies are applied in such small doses that they may just act as stimuli to prompt this reaction.
7. If a remedy has no specific relation to the individual reaction of the patient, it will not cause anything to happen, and there will be no side-effects. Small doses can only act with sensitised self-activating organisms.

### Causal Mechanics versus Teleology

These two basic attitudes towards the patient may explain, categorically, the differences of appearance between conventional medicine and homeopathy. The conventional view draws on paradigms of technical sciences and engineering, such as materialism, natural laws, and reproducibility. The homeopathic view, however, rests on a teleological perspective on living beings. Both paradigms at first glance may seem to be reasonable, even though opposing and contradictory.

By means of a philosophical analysis, it may however be shown that, irrespective of its plausibility and prevalence in modern societies, causal-mechanic and functional thinking may not be the highest and most comprehensive level of causality, but it may instead be teleological thinking that encompasses all other forms. 10 To make this point clear, a short but helpful digression to Aristotle may be of use: Contrary to our present-day understanding of the terms cause and effect, Aristotle distinguished four classical causes which may be exemplified by referring to the question, for example, ‘Why does a house exist?’.

Typically, there are four levels of answers. The house stands, first because it consists of stones and timbers or the like (called the *causa materialis* , because it mentions the physical matter as the cause), second because it was constructed by craftsmen (the *causa efficiens* ; it addresses the forces who built it), third because it was designed by an architect ( *causa formalis* ; it denominates the plan according to which it was built in a prescribed shape), and fourth because it was intended and desired by the home-builder ( *causa finalis* ; it states the wish of the owner). The fourth cause may prove to be of most importance, because without it the other causes (material, effective, formal) may never have been realised or relevant at all. 11

In analogy, the methodical restriction on the part of natural science and conventional medicine on lifeless objects, such as molecules, enzymes and other particles (and their interaction), on general laws of nature, and on the technical and pharmaceutic manipulation of the engine of the body, may be useful and correct—at the level of craftsmen, engineers and mechanics. However, to understand what is good, what is healthy, what is desirable for a certain individual, knowledge at this level may prove to be insufficient.

In fact, the art of medicine may require a higher, encompassing level of reasoning, namely teleological thinking. Only when patients are perceived as subjects—i.e. individual living beings who are striving for their integrity, interacting with their environment, and reacting to stimuli teleologically (that is with the purpose of self-preservation) may concepts such as health, illness and healing make sense at all.

Insisting on the need and indispensability of this higher, regulative, level of teleologic thinking does not mean, of course, that the categorically lower, reductionistic, level of conventional medicine would be wrong or refuted. The endeavour to find causal mechanisms or cybernetic circuits within the human body, and devices to manipulate them, has its merits and significance. For Plato and Aristotle, however, the mere capacity to change the state or function of the human body by medicinal means only ranks among the preliminary skills of a physician, whose real competence would be the faculty to know how to give to which patient what remedy, at what moment, and in what dose—in regard to his health. 12 13 14 Human medicine may never progress on the level of natural science alone, ignoring the other, higher dimensions of human beings that may only be apprehended by human or moral sciences: the humanities, including philosophy.

## Theory of Medicine

These findings from the science of philosophy may be perfectly complemented by the science of theory of medicine, where scientists emphasise that medicine may not be conceived as an applied natural science, but conceptualised as a practical science *sui generis* , i.e. a practical science in its own right. Contrary to natural science which, according to its self-conception, is primarily focused on cognition and knowledge and only secondarily reflects on future possible applications, the basis of practical medicine is always in terms of the mandate of the patient to the physician to act for his or her benefit. The starting point, as well as the ultimate justification of the physician's activities, is the well-being of his/her client, the patient. Every action, cognisance and science has to be related to this aim.

The difference in principle between a practical science (such as medicine) and a cognitive science (such as natural science) may be illustrated by a comparison of Acting and Knowing 15 16 :

- Action may have to be legitimised. Knowledge only has to be verified.
- Action may be normalised, standardised, stipulated. Knowledge not: it can only be true or false.
- Action is obliging, binding, committing for a person. Knowledge not: it is true or false.
- Action may be allowed or prohibited. Knowledge is mostly of a statistical nature.
- Action is irreversible. Knowledge can be reversed, i.e. revised, discarded or updated.
- Practical action cannot be performed exactly. Knowledge has to be exact.
- Action cannot be performed partly or with a certain probability. Knowledge can have a certain probability.

This outlining of the fundamental differences of the categories Acting and Knowing may elucidate again the necessity that a practical science (such as medicine) has to develop its own methodology, constituting itself as a science *sui generis* , because it can never rely entirely on an external mere cognitive science (such as natural science). Hence, it is a logical fallacy to consider medicine an applied natural science.

## Homeopathy versus Economisation of Medicine

In contrast to conventional medicine, classical homeopathy has basically been aware of these problems and therefore was, from the beginning, conceived as an art of healing. Although more than conversant with all the preliminary sciences of medicine, such as chemistry, physics, botany, anatomy and physiology, Hahnemann did appreciate their achievements, but never allowed their predominance in medicine. In respect of healing, he found that they cannot really help, and therefore—through a methodical and rational approach, being at the same time unbound to the reductionist level of natural sciences—he developed his own and self-sufficient medical science. 17 18

From the perspective of various human sciences (such as philosophy and theory of medicine), it may indeed appear that homeopathy fulfils the criteria of scientific medicine *better* than conventional medicine. Hence, the imposition to prove the scientificity of homeopathy by subjecting it to the framework of clinical trials, i.e. tools that may mainly make sense within a reductionist horizon of conventional medicine, may be counterproductive.

But how is it that this scientific insight is almost unknown within the medical establishment or by society at large?

To illuminate this paradox, the social sciences are challenged. Obviously, it may concern the sciences of sociology of knowledge, sociology of science, and sociology of medicine, to disclose how, in Western democratic societies, interpretive sovereignty comes about. It may be shown that any idea being true is not necessarily equivalent to it being accepted, let alone being meaningful and widespread. As a rule, ideas only then become part of the general knowledge of a community or society if they are in line with its basic convictions and values. 19 20 21 22

Insofar as today's moral values seem to be outplayed by economic values, it may be the field of the historical and economic sciences, such as history of economics as well as cultural studies, to detect the influence of economy on virtually every part of our life and culture, including science and medicine. As a matter of fact, in the long history of money, from first coinage and physical circulation to the invention of bills and stock exchanges to uncovered bonds, banknotes and plastic cards, monetary thinking played and plays an ever increasing role in all modern civilisations. 23 24

Thus, money has become the primary goal and incentive of everybody's daily efforts. It is the pattern in which we are socialised from childhood, and therefore has become the form in which we are conditioned to think (money as a form of thinking). 25 Since money transforms everything it touches into a commodity and can be accumulated by trading with commodities, the intellectual vision of man has continually become narrower. As a result, people today tend to look at everything, even medicine, in terms of commodities (called commodification). 26

## Natural Science and Conventional Medicine

At this point, the sciences of the history and theory of science may be needed to provide the link to the so-called scientific revolution in the 17th century when natural scientists developed the modern reductionist standards, such as generalisation, quantification, and reproducibility. 27 History of medicine may then show how, from the 19th century, this new methodology has brought forth many medical discoveries that could be perfectly marketed, such as chemotherapy, vaccinations, X-ray appliances, or the like. 28 29 In fact, markets are interested only in things that can be generalised, quantified, reproduced, etc. From an economic perspective, it appears completely consistent that conventional medicine has limited its scope to material causes of diseases detectable by technical devices, general diagnoses, statistics, and reproducible standard treatments—anything else cannot be marketed anyway. The blind spot in this approach, however, is the missing evidence on whether this machinery of commercial medicine, progressing under the euphemism ‘natural scientific medicine’, has anything (and if so, to what extent?) to do with the physician's task to heal or take care of the patient. Certainly, for methodological reasons, a reductionist wheelwork may on its own never be capable of realising dimensions such as a physician's task or the need of a patient, let alone of measuring or evaluating it.

## Hahnemann's Merit

Viewed against this background, Hahnemann's approach may appear all the more courageous—a beacon in a storm, as he had to swim against the tide, i.e. struggle against the rising mega-trend of economising medicine at the expense of the patient. But, guided by his strict moral values, his rational notion of God, his conviction of a higher calling of man, and his benevolence for his human brethren, his predominant concern was to help the patient. Under this premise, homeopathic theory and practice may prove to be perfectly consistent, beneficial, and scientific. 30

The aim of improving homeopathy's scientificity, therefore, should be attempted from within these constitutional conditions rather than from outside by means of tools that arguably were contrived by defiers of homeopathy with verifiable anti-homeopathic preconceptions. After all, the double-blind randomised clinical trial was introduced and pushed to become the ‘Gold standard’ in medical science—from the Cornell Conference on Therapy in 1946 onwards, under the driving force at Cornell University, Harry Gold 31 —amidst an agenda underpinned by the insinuation that ‘the enormous success of homeopathy’ was based on nothing but a ‘powerful placebo’. 32 33 34 35 36 37

## Discussion

Prompted by some of the theses, critical questions may arise. People may for instance wonder what is meant by, and how multidisciplinary research in the history of homeopathy may be achieved. A short answer may be: by opening up one's view, and taking into account the insights of neighbouring disciplines. Up to now, as a rule, either historians of other fields were unaware of homeopathy or historians of homeopathy were unaware of the findings of their colleagues from other fields. Instead, a multidisciplinary history of homeopathy would comprehend and consider as many perspectives as possible from any science on this topic.

For example, in the 1960s the historian of politics Quigley had already demonstrated the enormous influence of the development of capitalism (from commercial capitalism to industrial, financial and monopoly capitalism to pluralist economy) on the world's history and civilisation. 38 In the 1930s, 1960s and 1970s, the historians of science Fleck, Kuhn, and Feyerabend had pointed out the generally underestimated role of extra-scientific influences on the shaping and development of scientific paradigms, and emphasised that science may be considered to be a social process rather than a pure quest for truth. 39 40 41 42 In the 1990s, the historian of medicine Kaptchuk showed how much effort (including persuasiveness, propaganda and cheating) after World War II was needed by an academic elite to introduce to a reluctant medical profession the new tenets of a putative powerfulness of placebo, and the need of blind assessment, and concluded that their final adoption ‘had as much to do with shifting political, moral, and rhetorical agendas and technical research design issues as with scientific standards of evidence’. 32 33 In the 1980s, the historian of science Kohler came to a similar confirmation: ‘One cannot distinguish purely technical aspects of ideas from their role as political strategies in the competition for resources. … Ideas are judged not only for their truth value but also for their utility in discipline building’. 43 In the 2000s, the historian of economics Brodbeck pointed out in great detail that money (as a form of thinking) had developed in the course of history to determine contemporary rationality to such a degree that even within established schools of economics its influence on theory building is uncritically being overlooked. 25

As to the history of homeopathy, until now only few attempts have been made to consider and overview these and many more explosive insights from other disciplines. 44 45 46 Given the plethora and complexity of relevant perspectives, however, such an agenda of a critical evaluation of homeopathy's place in the history of medicine, science, culture and economics may not be easily accomplished by a single scientist, but only by means of multidisciplinary cooperation.

However, this leads to another point that may be difficult to realise: it may be of paramount importance to be clear on the direction in which future research may most appropriately be conducted. Hence, to call for multidisciplinary research may not be trivial but be a first decisive step concerning everybody. Collecting and scientifically processing data, on the one hand, and making sense of them or reflecting on final goals of research, on the other hand, are different things. To consider this difference in all its bearings, however, a philosophical standpoint may be needed.

At least since the seminal works of Kant it may be known by philosophers of nature that to study phenomena of the living (contrary to purely physical things!) scientifically, i.e. within the borders of our experience and categories of reason, we need to resort to so-called regulative ideas, such as the principle of teleology. 47 48 The crucial point in appraising this proposition correctly, however, is that it is not claimed that something like purposiveness ‘exists’ plainly in an ontological sense, but simply that, to make sense of the many empirical data observable in living objects, we are compelled by reason to look at living beings ‘as if’ they were acting according to a purpose. 49 50

To be sure, teleology as the regulative idea with which (mechanical and other) causal chains within creatures may be coordinated clearly transcends strict physical science, i.e. the science of non-living matter. Therefore, it neither may be proven nor disproven by the latter. Just as, in the coronavirus crisis, decisions of politicians may take into account and be based on pondering certain scientific facts and data against others but cannot themselves be determined or proven or unproven by those data, a sound consensus on a scientific agenda and methodology in the history of homeopathy may be a standpoint that cannot be deduced from single physical or mathematical sciences, but only emerge from a multidisciplinary effort by natural, cultural, and philosophical scientists.

The same may apply to Hahnemann's concept of a ‘vital force’. As a teleological principle, i.e. a regulative idea, untainted by any claim of physical existence, it can never be detected by a measurement tool and neither be ‘disproven’ for example by the first law of thermodynamics or the like, nor directly ‘proven’ by empirical data. However, for a physician treating living humans it may be an indispensable tool and a basis for his/her art of healing.

Aristotle, apart from introducing the concept of teleology into philosophy, has enriched medical science (in a broad historical sense) by many more invaluable categories, and homeopathy may be well advised to follow his line of argumentation. 51 In addition, his unabated actuality may be also seen from his analysis of economics, which may help to clarify the critical relationship between Hahnemann and modern, predominantly (not exclusively) economy-based medicine.

As in the fifth and fourth century BC monetisation and commerce were already far advanced in the urban centres of Greece and India (and nowhere else in the world), 52 including all temptations of misuse, Aristotle was first to coin the terminological distinction between the art of *oikonomiké* and the art of *chrematistiké* , the former meaning domestic economy, qualified as natural ( *para physin* ) and limited, and the latter signifying the attempt of earning money as an end in itself, assessed as unnatural ( *kata physin* ) and unlimited. Aristotle's critique of the latter was that money, in its natural function being a means for the purpose of housekeeping, here becomes the supreme purpose itself and thus perverted. 53 This dichotomy persists today: money may either serve as an expedient to a higher end or it may rule by debasing everything and everybody to a means for the appropriation and augmentation of itself.

Against that background and backed by facts from the history of economics as well as his biography, it may be comprehended that Hahnemann, living some 200 years ago in Saxony, where industrialisation, commercialisation, and commodification were still in their infancy, could behave with much less economic bias or conflict of interest than any physician of the 21st century who lives in a world defined and determined essentially by capitalism, which by its very nature tends to subject as much activities of as many citizens as possible to the goal of making money and maximising profit. Of course, it may still be possible today to resist this all-pervasive pull, but it needs much more independent standing and critical consciousness than in the economically still more innocuous time of Hahnemann.

True, in 1800, even Hahnemann tried to ‘sell’ his newly discovered preventive remedy against scarlet fever ( *Belladonna* ), but only in exchange for a subscription to its associated booklet, and he stopped this plan before long due to lack of subscribers. In the same year he did sell a supposedly new chemical substance discovered by him ( *alkali pneum* ), but as it soon turned out that this was nothing but ordinary *borax* , he publicly admitted his embarrassing error and donated the entire revenue to a fund for the poor. 54 These are the only two cases in Hahnemann's lifetime where he might be charged by adversaries of having sold ‘secret remedies’. Considering the context, a time of dire financial need when the breadwinner of a 10-person family had to battle for their existence, it seems ridiculous if not impertinent to insinuate here greed for money. 55 In Aristotle's terms, Hahnemann may still be imagined as an instructive example of a righteous housekeeper ( *oikonomos* ), to be contrasted with the excesses of casino capitalism of modern times and its adverse effects on medicine.

This relates to one of the core theses of this paper: to understand homeopathy and its history comprehensively, i.e. from a non-reductionist perspective as broadly as possible, it may be crucial to take into account its founder's environment, in terms of the moral, cultural, scientific and economic spirit of the time. To grasp all the differences against today in depth and in principle, however, may be too large a task for a single scientist but rather require a multidisciplinary collaboration and sharing of insights between natural sciences and the humanities, including their respective historians. For example, for modern scientists without a special training in history or cultural studies, it may be difficult to see the tremendous socio-economic changes affecting the contemporary framework of science as easily as historians of economics etc. may do. Here, the real challenge may first of all be to create a consciousness geared towards the necessity of opening up towards other disciplines.

An abridged summary, just in headline points, may perhaps read like this: In Hahnemann's time science was less constrained by methodological standards, less separated from philosophy, theology and the arts, and less fixed on the present kind of rationality determined by money (money as a form of thinking). With the rise of industrialisation, commercialisation, commodification (the view on all things as commodities), etc. and their associated shaping and narrowing of rationality in terms of quantification, standardisation, reproducibility, etc., the humanities lost ground while the term ‘science’ was finally identified with empirical, statistical and computational science. Within this macro-economic historical framework, medicine similarly developed (very roughly speaking) from individualising naturopathic patient-side approaches towards general standard treatments by synthetic drugs marketed by international companies and sanctioned by methodological tools developed for serial production of commodities.

Into this context, historians of science, medicine and civilisation might also place the introduction of the RCT as the ‘Gold standard’ for, so to say, the scientifically sanctioned selection of desirable from undesirable drugs. As commented on above, its anti-homeopathy bias may hardly be overlooked: one of the arguments in 1946, to finally convince the conventional medical profession to adopt the new methodology of randomised blinding with placebo was: ‘The enormous success of homeopathy, where drugs are given in great dilution, in sugar pills, drugs so dilute that they could not possibly have any pharmacologic action, is a good example. Its success and therapeutic results are probably better than those in the case of some of the regular drugs that are given in huge doses by the rival practitioners. At least, it has demonstrated very clearly what can be done by placebo’. 35

As an irony of history, it was homeopaths, by complying with the very standards designed by their competitors to bring about their scientific refutation, who presented placebo-controlled clinical studies with positive results. However, these credulous efforts yielded little to convince their opponents and their preconceptions. This paradox may be explained by means of a mathematical formalisation, the Bayesian theorem, according to which the prior probability that a scientist assigns to an hypothesis determines the posterior probability he/she assigns to it after regard to a certain event. 56 So, for proponents of homeopathy with an *a priori* positive view of the possibility of homeopathy, a significant *p* -value of an RCT may be considered as proof, while for critics it may be negligible, because their prior probability is so low that even a result with a small chance of occurring accidentally cannot change it much.

Ultimately, pros will remain pros and cons will remain cons, almost independently of the result of any RCT. Hence, homeopaths may better be advised to refrain from hopes on a breakthrough of recognition by means of RCTs. Rather it may be prudent, for the sake of retaining a holistic and humanistic self-image, to keep a critical distance from its underlying rationality with its tricky focus on numbers.

Today, from the perspective of ‘brave new science’, it may be difficult to be aware what dimensions may have been lost or diluted in terms of putting oneself in a relation to the world. Encouraged by its omnipresent achievements, modern science tends to gauge and evaluate everything according to its measuring units and methodology. High-minded specialists even deem it possible to explore and explain phenomena previously handled as philosophical ideas, such as life, vital force or health, by new scientific models. Though exciting as this may be from the perspective of the single scientist, as soon as we would claim to be able to ‘explain’ what such objects really ‘are,’ or provide ‘definitions’ of life, health, healing, etc., we would commit a naturalistic fallacy. Just like that which is good may not be explained reductively in terms of natural properties, such as pleasant or desirable, 57 neither may life be explained, e.g. by ‘self-organisation and emergence’, 58 nor the vital force by a ‘quantised gyroscopic metaphor’ 59 nor health by ‘regulation’ or ‘self-organised criticality’. 60 61

To be sure, this paper was not intended to show how on the forefront of sophisticated scientific model-building homeopathy may be endowed with a modern scientific appearance, say in terms of complexity theory, whole systems research, or the like. 62 Rather, the emphasis here is on homeopathy being a practical art of healing or a medical science *sui generis* . Accordingly, the seven premises of conventional medicine and of homeopathy respectively are meant to outline the basic ideas and principles guiding practitioners in their treatment of real humans in real life. So, while homeopaths may typically consider their patients as individual subjects animated by a vital force, reacting to stimuli and to be cured by an allegedly dematerialised single remedy, conventional doctors may primarily see the general aspects of patients whose corporeal components may be examined physically and chemically and treated by several material drugs for each dysfunction.

On this understanding and with regard to ordinary practice, conventional medicine may indeed still be described in terms of mechanism, materialism and positivism, despite these being concepts of the 19th century. Even though the theoretical science of medicine may well be more advanced, the final common path of the practical (and economical) application of new discoveries may remain—as a result of the practical training at medical school—the general practitioner's materialistic view on the patient (in terms of commodification) and his/her prescription of biochemical substances or mechanical devices (in terms of commodities).

On the other hand, the homeopathic practitioner may still be adequately guided by the principles adopted by Hahnemann. Philosophically, these may be regulative ideas in the sense of Kant, being neither scientifically provable nor disprovable. From the perspective of theory of medicine, however, they may appear to be indispensable (and perennial) principles of any art of healing. Any loss of awareness of the artistic essence of homeopathy that has happened over two centuries may prove to be just another facet that a multidisciplinary history of homeopathy might be open to apprehend and integrate.

## Conclusion

To suggest that Hahnemann's homeopathy may be considered to be an exemplary scientific medicine (in a broad sense) and medical science *sui generis* does not mean that conventional medicine would not be scientific. Both may be scientific, but on different levels, with different methodologies, and driven by different interests. Disclosing that the machinery of conventional medicine may predominantly (i.e. not without exceptions) be driven by economic incentives, does not preclude that in many cases it may nevertheless be useful and of need. In fact, much health improvement has come from conventional medicine that is not financially driven. On the other hand, the fact that homeopathy is primarily committed to the patient's welfare does not preclude that in many cases it may be rewarding not only morally but also economically.

To comprehend in principle the charged relationship between a primarily (not exclusively) money-driven and a primarily (not always purely) humanitarian medicine, the two poles have been depicted possibly in a somewhat exaggerated form. This is simply to facilitate the perception of the antagonistic gravitational forces and omnipresent attractors to which in principle everybody is exposed today. Although in real life the majority of therapists and agents in the health care system may not represent absolute manifestations of one or another extreme, but rather are mixed types, it may well now be possible to determine anybody's position more precisely once the essential difference of the two approaches has been grasped.
